# A New Look to a Classic Issue: Reasoning and Academic Achievement at Secondary School

**DOI:** 10.3389/fpsyg.2018.00400

**Published:** 2018-03-28

**Authors:** Isabel Gómez-Veiga, José O. Vila Chaves, Gonzalo Duque, Juan A. García Madruga

**Affiliations:** Psicología Evolutiva y de la Educación, Universidad Nacional de Educación a Distancia, Madrid, Spain

**Keywords:** reasoning, academic achievement, executive processes, secondary school, learning

## Abstract

Higher-order thinking abilities such as abstract reasoning and meaningful school learning occur sequentially. The fulfillment of these tasks demands that people activate and use all of their working memory resources in a controlled and supervised way. The aims of this work were: (a) to study the interplay between two new reasoning measures, one mathematical (Cognitive Reflection Test) and the other verbal (Deductive Reasoning Test), and a third classical visuo-spatial reasoning measure (Raven Progressive Matrices Test); and (b) to investigate the relationship between these measures and academic achievement. Fifty-one 4th grade secondary school students participated in the experiment and completed the three reasoning tests. Academic achievement measures were the final numerical scores in seven basic subjects. The results demonstrated that cognitive reflection, visual, and verbal reasoning are intimately related and predicts academic achievement. This work confirms that abstract reasoning constitutes the most important higher-order cognitive ability that underlies academic achievement. It also reveals the importance of dual processes, verbal deduction and metacognition in ordinary teaching and learning at school.

## Introduction

Human thought involves the building of mental representations by integrating external and previously stored information, and their manipulation in a cognitive space: working memory (WM; Baddeley, 2007). A prime characteristic of thinking is hence its abstract nature. It always requires people to construct specific representations from the perceptual features of stimuli and individuals' knowledge and goals (Johnson-Laird, 2006). Moreover, higher order thinking abilities such as complex text comprehension, reasoning, and meaningful school learning are sequential. They consist of diverse component subtasks and demand that people keep their attention focused throughout the entire process. Besides the initial construction of representations, higher cognitive tasks require individuals to keep the goal of the task in mind, to shift from one sub-task to the next, to update representations by activating Long Term Memory (LTM) information, and to inhibit and discard irrelevant processes and responses. The fulfillment of these complex cognitive tasks demands that people activate all their WM resources in a controlled and supervised way (see, García Madruga et al., 2016).

The important role that higher order thinking abilities such as reasoning plays in knowledge acquisition and attainment at school has been addressed mainly from the perspective of the relationship between intelligence and academic performance (see, e.g., Sternberg et al., 2001; Deary et al., 2007; Vock et al., 2011). Classical psychometric theories have frequently defined intelligence, particularly fluid intelligence, as a capacity based on abstract reasoning (see Thurstone, 1938; Sternberg, 1985). Diverse authors have maintained that a crucial component of fluid intelligence is relational reasoning (see Cattell, 1987; Dumas et al., 2013), that is, the ability to identify and integrate the relationships between multiple mental representations. As a number of studies have shown, intelligence is probably the best single predictor of academic achievement in diverse subjects (Kuncel et al., 2001; Deary et al., 2007), particularly in mathematics-science subgroup of school subjects (Roth et al., 2015). The correlations found between fluid intelligence tests and academic achievement measured by means of scholastic tests are around 0.5 or higher (see, e.g., Neisser et al., 1996; Ones et al., 2004; Colom and Flores-Mendoza, 2007; Deary et al., 2007), whereas when the academic achievement measures are based on teacher grades the correlations tend to be around 0.5 or lower (see Soares et al., 2015). This evidence is in accordance with the results of studies that showed that increases in reasoning abilities were also accompanied by improved learning of classroom subject matter (Klauer and Phye, 2008). A possible explanation would be that fluid intelligence is associated with reasoning abilities (both inductive and deductive) involved in understanding and solving novel complex problems (Greiff and Neubert, 2014).

There is a relatively new theoretical approach on thinking and reasoning in which WM plays a crucial role: dual-process theories (see Sloman, 1996; Stanovich, 1999; Evans, 2008; Kahneman, 2011; Evans and Stanovich, 2013). These theories postulate the existence of two different types of thinking: System 1 (intuitive) and System 2 (deliberative). System 1 is fast, unconscious, associative and not dependent on WM. System 1 allows individuals to quickly access intuitive responses that can be valid but also a source of pervasive mistakes. On the other hand, System 2 is slow, conscious, controlled and strongly linked to reasoners' WM as well as their thinking dispositions or mental styles. WM is thus a defining feature of analytical System 2 processing. System 2 processing is required to solve complex thinking and reasoning problems, although this is not a sufficient condition for valid responses. Most dual-processing theories assume System 1 processing yields intuitive responses that subsequent System 2 deliberation may or may not modify. Stanovich et al. (2011) claim that deliberative reasoning requires overriding System 1. Overriding System 1 and activating System 2 demands an individual's executive control, as well as a propensity to think actively and resist the premature closing of problems. Executive control processes thus play a crucial role in analytical System 2 processes (see De Neys and Glumicic, 2008; Evans, 2009; Thompson, 2009). System 2 function relates to general measures of cognitive ability such as IQ (Intelligence Quotient), whereas System 1 function does not (Stanovich, 1999). Dual process theories of thinking have been mainly developed in deductive reasoning tasks and there is a lot of evidence for the relevance of both systems of thinking in propositional and syllogistic reasoning (see De Neys, 2006; Evans, 2008; Barrouillet, 2011).

Reasoning is a kind of thinking activity that has a precise starting point, a set of premises, and the goal of drawing a conclusion. In induction, the conclusion involves an increment in semantic information; whereas in deduction the conclusions do not involve any increase in semantic information (Johnson-Laird, 1988). Abstract reasoning, even the most elementary kind, is hence a complex phenomenon that requires that individuals follow a sequential process that includes various steps and tasks, and the passage from one to another. A second source of complexity comes from the need to temporarily store and update in WM the diverse representations needed to carry out a reasoning sequence. Indeed, individual differences in reasoning are substantially correlated with the amount of information learners can hold in WM while perform the required inferential reasoning task (Süß et al., 2002).

There are diverse theories of deduction, the two most important being that of “mental rules” and “mental models.” According to mental rules theories, people possess a set of rules, a sort of “natural logic” from which they reach a conclusion by following a sequence of steps (see Rips, 1994; Braine and O'Brien, 1998). The current work, however, has been planned and carried out according to the mental model approach. The mental model theory of reasoning postulates that when individuals face deductive problems they construct models or possibilities of the meaning of assertions consistent with what they describe (see Johnson-Laird, 1983, 2006; Johnson-Laird and Byrne, 1991). Mental models only represent what is true according to the premises, but not what is false. For instance, for the conditional “if p, then q” people might construct the following true possibilities: “p and q,” “not-p and q,” “not-p and not-q.” A main assumption of model theory concerns the crucial role of WM in deduction: representing and manipulating models in order to reach a conclusion entails cognitive work and effort. For complex deductive problems, for instance conditionals, several possibilities are true, but people think about as few alternatives as possible because of the limitations of their WM (Johnson-Laird and Byrne, 2002). Therefore, reasoners are likely to base most of their inferences from the initial and incomplete representation or models of the premises. The model theory's fundamental prediction is thus drawn from the number of models required for reaching a conclusion: the more models, the greater the problem's difficulty. When the number of models to be held in mind is reduced, reasoning improves (García-Madruga et al., 2001). An inferential conclusion is necessarily valid if it holds in all the models of the premises. Therefore, finding a valid conclusion to complex problems requires that individuals build complete representations of premises and validate initial conclusions by searching for counterexamples that can make them false. From a mental model perspective, García-Madruga et al. (2007) have highlighted the central executive processes as the crucial WM component in the explanation of propositional reasoning performance, as well as its relationship with the two systems of reasoning processes, System 1 and System 2. They found positive correlations between WM and reasoning responses that require high levels of mental word, and negative correlations between WM and reasoning responses that require low levels of mental work. Moreover, high WM individuals were more able to resist superficial responses and rely on the semantic process of constructing models of the meaning of premises from which they drown correct conclusions. Likewise, studies with syllogistic reasoning problems have borne out the crucial role of WM, particularly the executive processes (see Gilhooly et al., 1999; Capon et al., 2003).

In deductive reasoning, the process of connecting premises to the conclusion is ruled by logic. That is, the conclusions have to be necessary and consistent. The restrictions that rule deductive reasoning are hence metadeductive. Metadeduction involves an individual's capacity to reflect on one's own logical activity itself and to distinguish between logical validity and reality (see, e.g., Byrne et al., 1993). Metadeductive abilities include the implicit understanding of the logical system and its basic concepts of necessity and validity, as well as the explicit use of this knowledge by applying metalogical strategies, such as searching for counterexamples (Moshman, 1990). Metacognition has been proposed as an important basis for overrides of System 1 by System 2 (Thompson, 2009). In developmental terms, there is a gradual acquisition of metalogical understanding during preadolescent and adolescent years: from 11 to 12 years preadolescents begin to understand the concepts of necessity, consistency and the validity of logical conclusions (Moshman, 2004; Santamaría et al., 2013). However, complete explicit metalogical capacity only becomes possible in late adolescence and adulthood. In this work, we will use a Deductive Reasoning Test (García-Madruga et al., 2014a) that assesses both deductive and metadeductive abilities.

Dual process theorists agree that abstract and hypothetical System 2 processes can guide our reasoning on specific tasks toward normative answers, whereas System 1 processes have a mayor influence on everyday judgement. The role of dual processes of thinking in education, and particularly the capacity of the Cognitive Reflection Test (Frederick, 2005) to predict academic achievement, have been demonstrated by Gómez-Chacón et al. (2014) within the field of mathematics. According to these authors, the dual processes approach has direct application to mathematical learning. A teacher has to direct attention toward two basic educational objectives: firstly, the promotion of an in-depth understanding of mathematical concepts; and secondly, the inhibition of superficial processes and strategies that otherwise lead to error. Our view is that these two basic objectives are cross-sectional in education, so that they underlie academic achievement not only in mathematics, but across all the diverse school subjects.

The present paper addresses the relationship that abstract reasoning has with academic achievement in secondary school, and focus not only on classic fluid intelligence measures but also on two new measures of cognitive reflection and verbal deduction. We examine the underlying idea that other reasoning measures will be a relevant predictor of academic achievement, even beyond the predictive value of fluid intelligence measures. We argue that abstract reasoning processes are involved in most of the complex learning tasks students commonly face at school, and constitute an important higher-order cognitive ability that underlies academic achievement. In order to extend and clarify this supposition, the main goal of this study was to investigate the relationship between two new reasoning measures, one mathematical (Cognitive Reflection) and the other verbal (Deductive Reasoning), and a third classic visuo-spatial reasoning measure that evaluates fluid intelligence, as well as test their capacity to predict academic achievement in adolescence. In spite of the obvious differences in contents and materials, all of them involve the construction and manipulation of abstract representations in a sequential process that requires supervision and control. Thus, we provide a new estimate of the association between cognitive ability and education by having multiple reasoning tests as predictors of academic achievement at secondary school. Participation of an adolescent sample seemed particularly relevant, as reasoning skills are increasingly important during this developmental period (Barrouillet, 2011), when learning activities become more complex. In order to assess this view, we used three diverse kinds of reasoning tests, as we shall describe in more detail in the Methods section: Cognitive Reflection Test (CRT; Frederick, 2005), DRT (García-Madruga et al., 2014a) and Raven's Progressive Matrices Test (RPMT; Raven, 1941, 1962; Raven et al., 1995). The academic achievement is the criteria for assessing students learning outcomes.

The Cognitive Reflection Test (CRT; Frederick, 2005; for a review, see Campitelli and Gerrans, 2014; for a metaanalysis, see Brañas-Garza et al., 2015) assesses an individual's ability to use System 2 processes and resist the tendency to give an immediate response (System 1). Frederick (2005); Toplak et al. (2014) found that many people show a tendency to give the fast incorrect answers that are automatic, superficial, compelling, without thinking deeply enough. Frederick's problems are hence difficult and people who give the incorrect responses tend to underestimate their difficulty. Prior research has also evidenced that individuals who perform well tend to perform also well at other general ability tests, and tend to avoid biases in decision-making (Campitelli and Labollita, 2010; Toplak et al., 2011). Therefore, we expected a high rate of erroneous intuitive responses and a significant positive correlation between intuitive responses and the rating of the difficulty of the problems.

The DRT (García-Madruga et al., 2014a) assesses both deductive and metadeductive abilities in propositional and syllogistic reasoning. According to mental model theory, most of the deductive problems included in the test (11 out of 15) require the construction of multiple models, that is, require the use of System 2 reflexive thinking. The problems were designed so that they covered a range of difficulty (see Johnson-Laird and Byrne, 1991, for an analysis of tasks in terms of mental models). We hence expected that most of the problems would be difficult and predicted a close relationship between performance in CRT and DRT.

In order to have a measure of abstract non-verbal reasoning, in this work we used the RPMT (Raven et al., 1995) that evaluates fluid intelligence. The RPMT assesses a participant's ability to solve new problems by perceiving relationships, inducing rules and completing abstract analogies; in other words, by solving diverse relational reasoning tasks that are carried out in working memory, and governed by the executive processes of “assembly and control” (Carpenter et al., 1990). The more difficult problems entail more rules or more difficult rules. Erroneous responses can be yielded by System 1 processes, that is, by a superficial or incomplete kind of reasoning that leads one to give intuitive responses that agree only partially with the rules governing the search for the correct responses. We hence also predict a close relation between performance in CRT and Raven test.

Apart from the general expectations concerning the results in CRT, we expected positive correlations between cognitive reflection, deductive reasoning, and Intelligence measures, given that there are common cognitive, metacognitive and WM's executive processes that underlie them. Likewise, we predicted that the three reasoning tasks would correlate with academic achievement. In particular, the correlations between erroneous intuitive responses (System 1) and the ratings of difficulty in CRT with accuracy measures of reasoning (System 2) and academic achievement should be negative. Our second hypothesis includes the main prediction that cognitive reflection, deductive reasoning and intelligence measures should predict a relevant amount of variance of academic achievement. Regarding the CRT, recent findings indicate that it is a predictor of rational thinking performance (Toplak et al., 2011, 2014). Our approach maintains that CRT provides a way to assess a main control executive function: the ability to supervise and inhibit cognitive processes and responses. Therefore, we expected that CRT would be able to predict a relevant amount of variance of deductive inferences, metadeductive inferences and intelligence.

## Method

### Participants

Fifty one science students aged between 15.3 and 17.7 years (*M* = 15.97; *SD* = 0.45) participated in the study. They were recruited from a 4th grade (2nd cycle of compulsory secondary education refers to 3rd and 4th grades) of a state secondary school in Fuenlabrada (Madrid, Spain). They were 19 girls and 32 boys (62.35%) distributed in two class-groups (Group A: 27 students, 11 girls; and Group B: 24 students, 8 girls). We chose to select science students because scholastic achievement in sciences usually depends more heavily on reasoning abilities than other subjects like humanities, presumably because those subjects are more hierarchically structured (Deary et al., 2007). The school was selected to represent the public educational secondary schools from a medium sized urban area with varied socio-economic classes. Students who did not complete some of the tests and those whose families did not authorize participation in the research were excluded (13) from the final sample (*n* = 51). The sample includes three repeater students that were not excluded from the sample in order to preserving natural class-groups.

### Tasks and measures

#### Cognitive reflection test (CRT; Frederick, 2005)

The CRT assesses an individual's disposition and aptitude to be reflective when faced with finding the solution to text-based mathematical reasoning problems. For instance, one of the problems is as follows: “*A bat and a ball cost 1.10 $ in total. The bat cost 1 $ more than the ball. How much does the ball cost?____cents*.” The mathematical knowledge required to check the accuracy of the intuitive response is rather simple. However, Frederick (2005); Toplak et al. (2014) found that many people show a tendency to give the fast incorrect answers that are automatic, superficial and compelling —10 cents—. In other words, wrong answers are typical System 1 responses. The correct answer —5 cents— requires System 2 reasoning: only individuals who overcome the System 1 response, deliberately think more in depth and construct a more complete representation can solve the problems. As Campitelli and Gerrans (2014) evidenced, it is a test of cognitive reflection and not just a numeracy test.

A Spanish version of this test was used. Participants are asked to solve three numerical problems. There is no time limit to solve the problems and no alternatives are provided for the participants to choose. Afterwards, participants are also asked to evaluate the percentage of their classmates that will be able to give a correct solution to each problem. The solutions to each problem and the estimated percentages of correct solvers have to be written down in a booklet. We have three kinds of measures the CRT for each problem: correct (System 2) responses, intuitive (System 1) responses, and a rating of the problem's difficulty. The total score in CRT is calculated as the number of correct answers. Reliability of Cronbach alpha range between 0.60 and 0.73 values (see, Campitelli and Gerrans, 2014).

#### Deductive reasoning test (DRT; García-Madruga et al., 2014a)

The DRT is composed of 15 items and covers four types of problems: propositional deductive and propositional metadeductive inferences, and syllogistic deductive and syllogistic metadeductive inferences. All the problems were presented with concrete materials and in a familiar and daily context. The Cronbach's alpha in DRT was 0.79.

The propositional deductive inferences were four: two conditional problems, e.g., “If p then q” (Affirmation of Consequent, AC: q then q; and Denying of Antecedent, DA: not-p, then not-q) and two inclusive disjunction problems, “p or q or both” (one affirmative and one negative). In these problems, participants are asked to evaluate the possible conclusions of these four inferences. The problems require the construction of multiple models.

The propositional metadeductive inferences were three truth-table problems in which participants had to analyze the consistency of three problems that consisted of a conditional statement and an assertion. In the first problem, the assertion matched the first initial model of the conditional (p and q) and most of participants should choose the correct response. The second problem required one to construct the second conditional model in which antecedent and consequent are negated (not-p and not-q). Finally, the last problem demanded the construction of the third and most difficult model of conditional in which the antecedent is negated but the consequent is affirmed (not-p and q).

The syllogistic inference task required participants to generate and write the solution to five syllogisms. These syllogisms included the combinations of the four kinds of premises: universal affirmative (A: All X are Y), universal negative (E: No X are Y), particular affirmative (I: Some X are Y) and particular negative (O: Some X are not Y). The syllogisms were of different levels of difficulty. The first syllogism was the easiest: it was the only one-model syllogism. The rest of the syllogisms were multiple-model syllogisms which required the construction of two or three models. Two of the multiple model syllogisms had no valid conclusion (N). Finally, in the syllogistic metadeductive necessity/possibility task, reasoners had to decide if a given conclusion in three syllogistic problems is necessarily true, possible, or impossible. In this case, the first problem was the most difficult.

#### Raven's test of intelligence (RPMT; Raven et al., 1995)

The RPMT consists of sixty visual analogy problems. Participants have to identify the relevant features of an array of visual elements and then choose from among eight response alternatives arranged below the matrix which one is the correct element that has to be selected. Each problem consists of a 3 × 3 matrix (9 cells) that contains figural elements, such as geometric figures and lines, in which an element (bottom right position of the matrix) is missing. Participants have to look across the rows and then look down the columns to discover the rules that govern the presentation of the diverse figural elements and then use the rules to determine the missing element. Each problem presents a matrix with a missing element. A Spanish version of this nonverbal test was used. The Cronbach's alpha was 0.75.

#### Academic achievement

Participants' academic achievement measures were the students' achievements of content knowledge learned at the end of school terms; that is, the student's final numerical scores obtained in the seven basic subjects that include Language (Spanish), Biology, Physics, History, English, and two subjects of Mathematics. From the specific academic scores, we calculated two overall scores: Overall Math is the mean of the scores in the two mathematics subjects and Overall Achievement is the mean of the other disciplines (without Mathematics). The academic scores were obtained from the school database, but all data are confidential and anonymous. The students' scores were given by university qualified and professional teachers in the diverse subject matter. The numerical score includes performance on theoretical, practical and attitudinal contents. Teachers evaluate their students using a grading system that ranges from 1 to 10 points in each subject (5 to 10 = approval).

The use of teacher final class grades as summaries of students' academic achievement poses some difficulties and possible biases concerning the validity and reliability of grades for communicating meaningful information about students' academic progress (see, Allen, 2005). Nevertheless, the assessment of academic performance carried out by teachers in our study was specifically centered on the acquisition of knowledge within each of the different disciplines by giving each student a numerical score. According to Roth et al. (2015), school grades are a good measure of academic achievement since they include information on scholastic performance over a wide period of time, based on different sources (e.g., student's motivation) and are less prone to error than other kind of specific achievement tests. Besides, a number of researchers have used academic achievement as measures on academic achievement (e.g., Kuhn and Holling, 2009).

### Procedure

Participants were tested in one single session lasting 90 min. Each class-group completed the three reasoning tests in their classroom with a short break between them. The order of presentation of the tasks in group A was: RPMT, CRT, and DRT. In the group B the order was the opposite. The three tests were presented to students in specific booklets. Prior to data collection, permission was obtained from parents, teachers and students. Parents were informed that participation in the study involved completing a set of reasoning tasks, which were administered by the researchers in groups. The study and overall procedure were approved by the UNED ethics committee.

### Data analyses

The analyses were performed with the Statistical Package for the Social Sciences (IBM SPSS, 2010). No missing values were registered. Preliminary data screening indicated that the scores on reasoning and achievement measures were reasonably normally distributed. Because the outliers were not extreme, these scores were retained in the analysis. First, the percentage of correct and incorrect responses were calculated. To compare patterns of difficulty in the reasoning tasks, paired-samples *t*-tests were conducted. Second, Pearson bivariate correlations were performed to assess the relationship between cognitive reflection, deductive reasoning, intelligence and academic achievement measures. The strength of the correlations was classified according to the criteria suggested by Cohen (1988): a value of 0.10–0.29 is small; 0.30–0.49 is medium, and 0.50–1.00 is high. Third, to examine the predictive role of variables, the method of standard multiple regression analysis was conducted; that is, all predictor variables were entered in one step. To determine which of the reasoning predictors accounted for most of the variance on the academic achievement scores, we performed two standard multiple regression analyses, one to assess the predictive power of reasoning ability on math achievement, and another one to assess the predictive power of reasoning variables on overall achievement. These analyses included variables that were significantly correlated. In addition, three standard multiple regression analyses were conducted to evaluate how well intelligence, deductive inferences and metadeductive inferences could be predicted from CRT measures. Given that in the CRT correct and intuitive responses practically mirror one another, we introduce in our regression analyses either one or the other, but never both of them.

## Results

Below are presented the results structured according to the descriptive statistics of reasoning variables and objectives of the present study.

### Descriptive statistics and comparisons

The results of the diverse measures in the cognitive reflection task (CRT) can be observed in Table 1. The results of the CRT agreed with our expectations and Frederick's (2005) predictions: the CRT was rather difficult, there were more wrong intuitive (58%) than correct responses (33%; *p* < 0.05, Cohen's *d* = 0.64). Participants also clearly underestimated the difficulty of the task. That is, they considered that 76% of their classmates would be able to give correct responses, well above the total percentage of correct answers.

**Table 1:** The percentages of correct and intuitive responses, and the ratings of difficulty for each of the problems in the cognitive reflection test.

**Problems**	**Correct responses (%)**	**Intuitive responses (%)**	**Difficulty ratings (%)**
1. The bat and the ball	29	63	84
2. The machines	41	57	76
3. The pond and the lily pads	29	57	69
Overall	33	58	76

The results of the DRT can be observed in Table 2. The percentage of correct responses in multiple model problems was reliably lower than in one model problems (40 and 89%, respectively; *p* < 0.01, Cohen's *d* = 2.23). There was a reliable pattern of decreasing difficulty in the four deductive reasoning tasks: the syllogistic construction task was reliably more difficult than the propositional evaluation task (*p* < 0.01, Cohen's *d* = 0.78), which was more difficult than the propositional truth table task (*p* < 0.01, Cohen's *d* = 0.62), which was later found to be more difficult than the syllogistic necessity/possibility task (*p* < 0.01, Cohen's *d* = 0.37). Likewise, on the whole, deductive inferences were clearly and reliably more difficult than Metadeductive Inferences (*p* < 0.01, Cohen's *d* = 1.51).

**Table 2:** The percentages of correct responses (Standard deviation) for each of the problems in the Deductive Reasoning test.

**Problems and statements**	**Correct responses**	
**PROPOSITIONAL EVALUATION TASK**			
Conditional Inferences	DA	35% (0.48)
Inclusive Disjunctions	AC	25% (0.44)
	Aff.	69% (0.47)
	Neg.	78% (0.42)
	Overall Task	52% (0.27)
**METADEDUCTIVE TRUTH-TABLE TASK**			
If p then no-q	p q^*^	94% (0.24)
If p then q	not-p q	82% (0.39)
	not-p q	24% (0.43)
	Overall Task	67% (0.20)
**SYLLOGISTIC CONSTRUCTION TASK**			
	AI-I^*^	84% (0.37)
	IA-N	2% (0.14)
	EI-O	43% (0.50)
	EA-O	8% (0.27)
	OO-N	31% (0.47)
	Overall Task	34% (0.19)
**METADEDUCTIVE NECESSITY/POSSIBILITY TASK**			
	Possible	45% (0.50)
	Possible^*^	90% (0.30)
	Necessary^*^	88% (0.33)
	Overall Task	75% (0.23)
Deductive problems		42% (0.19)
Metadeductive problems		71% (0.19)
Overall correct responses		53% (0.17)

* *= one model problem*.

In the RPMT the mean of correct responses was 52.75 and the standard deviation was 3.97 (Max. = 60, Min. = 42). These results show the participants have a high level of fluid intelligence in comparison with their age group (*M* = 48).

### Interrelationships between variables

Table 3 presents the correlations between the mean measures. As can be observed, our predictions included in the first hypothesis were clearly confirmed. The correlations between CRT (correct responses), DRT (deductive and metadeductive inferences) and Intelligence measures were positive and significant. Likewise, accuracy in the three reasoning tasks correlated in reliable way with the two Academic Achievement measures. In DRT, the correlations between multiple and one model problems with Overall Math were: *r* = 0.48 (*p* < 0.001) and *r* = 0.12 (*p* > 0.1), respectively; and with Overall Achievement, the correlations were: *r* = 0.46 (*p* < 0.001) and *r* = 0.14 (*p* > 0.1), respectively.

**Table 3:** Pearson correlations between Cognitive Reflection Test (Correct and Intuitive responses, and Ratings of Difficulty), Intelligence (RPMT), Deductive and Metadeductive Inferences, and the two Academic Achievement measures.

***n* = 51**	**1**	**2**	**3**	**4**	**5**	**6**	**7**	**8**
1. Reflection Correct	1	−0.94^**^	−0.62^**^	0.45^**^	0.46^**^	0.57^**^	0.29^*^	0.33^**^
2. Reflection Intuitive		1	0.69^**^	−0.47^**^	−0.47^**^	−0.55^**^	−0.35^**^	−0.39^**^
3. Difficulty Ratings			1	−0.33^**^	−0.39^**^	−0.54^**^	−0.37^**^	−0.38^**^
4. Intelligence				1	0.27^*^	0.33^**^	0.41^**^	0.37^**^
5. Deductive Inferences					1	0.60^**^	0.48^**^	0.44^**^
6. Metadeductive Inferences						1	0.29^*^	0.28^*^
7. Overall Mathematics							1	0.89^**^
*M* = 5.58; *SD* = 2.09								
8. Overall Achievement								1
*M* = 6.54; *SD* = 1.57								

* p < 0.05;

** *p < 0.01; unidirectional*.

Finally, it may be noted that, as expected, the correlations between erroneous intuitive responses and the ratings of difficulty in CRT with accuracy measures of reasoning and academic achievement were all negative and significant (range between 0.35 and 0.69 values).This finding indicates that the underestimation of difficulty was higher in participants that gave intuitive responses.

### Predictive power of variables

Our second hypothesis claimed the predictive ability of the reasoning measures (Cognitive Reflection—either difficulty ratings or intuitive responses—, Deductive Inferences and Intelligence) on both Academic Achievement scores. Table 4 shows that CRT, DRT and Intelligence measures significantly predicted relevant amounts of variance of the two measures of Academic Achievement: Overall Math and Overall Achievement. The results satisfied our expectations as follows: 33% of the variance of Academic Achievement in Mathematics and 28% of the Academic Achievement in the remaining subjects—Spanish Language, Biology, Physics, History, English—were explained by the abstract reasoning measures. The reliable variables were Deductive inferences and Intelligence in the former, and only Deductive inferences in the overall achievement measure (without Mathematics).

**Table 4:** Regression analyses of Intelligence, Deductive Inferences and Cognitive Reflection (either Difficulty Ratings or Intuitive Responses) on the two Overall Achievement measures (Overall Math and Overall Achievement without Math).

**Dependent variable**		****R*^2^***	***F***	***B***	***Beta***	***Sig*.**
*Overall Math*		0.33	7.410^***^			
Intelligence				0.14	0.26	0.04
Deductive Inf.				3.81	0.34	0.01
Diff. Ratings				−0.01	−0.14	0.28
*Overall Achievement*		0.28	6.014^**^			
Intelligence				0.10	0.22	0.11
Deductive Inf.				3.11	0.32	0.02
Intuit. Resp.				−0.60	−0.13	0.39

** *p < 0.01*,

*** *p < 0.001; unidirectional*.

Additional multiple regression analyses confirmed that the CRT measures were able to reliably predict relevant amounts of variance of Intelligence (RPMT), deductive and metadeductive inferences (DRT). As confirmed by regression results, Intuitive responses and Difficulty Ratings scores predicted 23% of the variance in Deductive inferences, *F*_(2, 48)_ = 0.13, *p* < 0.01, and fluid Intelligence measures, *F*_(2, 48)_ = 6.92, *p* < 0.01, of which only the Intuitive responses variable (*B* = 0.18, *Beta* = 0.39, *p* < 0.05; *B* = 4.61, *Beta* = 0.47, *p* = 0.05, respectively) was reliable. The amount of variance of the Metadeductive inferences explained by Correct responses and Difficulty ratings raised to 37%, *F*_(2, 48)_ = 11.66, *p* < 0.001. In this case, although the Difficulty ratings score is close to reaching significance, only the correct responses score was reliable (*B* = 0.19, *Beta* = 0.40, *p* < 0.05).

## Discussion

In order to solve new and complex intellectual problems individuals have to think in an active and deep way, using all their WM resources and applying their main executive processes. In this paper, we have investigated abstract reasoning in 15–17 years old students in their final year of Secondary school, using three different reasoning tasks: the CRT, the deductive reasoning task and the fluid intelligence test. Our key predictions concern the relationships between the three kinds of reasoning measures and their capacity to predict academic achievement. With respect to this, we would like to make two main points: first, this study confirms the trend noted in previous research regarding the relationship between reasoning and academic achievement; and second, the present novel findings revealed the relevance of verbal deductive reasoning to predict academic achievement, even beyond a visual fluid intelligence measure.

We provide new evidence on the interplay between the three cognitive variables. As claimed by our first hypothesis, the inter-correlations were all significant and in the predicted direction. Furthermore, on the whole, the magnitude of predicted correlations was medium to high, in particular, the correlations between correct and intuitive responses in the CRT with Intelligence (RPMT), Deductive and Metadeductive inferences (DRT). These results confirm our theoretical conception regarding the tight relationships between the diverse kind of reasoning studied in this paper, as well as the common cognitive, metacognitive, and executive processes that underlie them. An interesting, but not unexpected, result is the high correlation between Cognitive Reflection and Metadeductive Inferences. The greater predictive ability of the CRT on Metadeductive inferences would be explained by the increased involvement of the metacognitive component in the metadeductive tasks. Thus, the pattern of results supports their common metacognitive nature and gives support to the important role that metacognitive processes may play in determining the interventions of System 2, as claimed by Thompson (2009), Thompson et al. (2011). It is interesting to note that, on the whole, the results confirm the relevant role that executive and metacognitive processes play in both verbal and visual abstract reasoning (García-Madruga et al., 2007).

The CRT mainly measures the propensity or willingness to be reflective, to think analytically despite having what initially appears to be a suitable response (Pennycook et al., 2015). According to our view, this thinking disposition is directly related with executive functioning, since thinking analytically implies the ability to supervise and inhibit cognitive processes and responses. The present findings confirmed that, as expected and found in previous studies (Brañas-Garza et al., 2015), most of the participants replied with the wrong intuitive responses in the CRT and also underestimated the difficulty of the task: there was a high correlation between both measures. Only a third of participants were able to resist and override the immediate spontaneous answer and keep thinking to reach the correct one. The analytic thinking carried out by these participants not only allowed them to reach a correct conclusion, it also increased their metacognitive awareness of the difficulty of the problems. Thus, the correlation between correct responses and difficulty ratings measures was highly negative. As explained by Frederick (2005), the correct answers produced by System 2 involve cognitive and motivational effort as well as concentration, and for this reason require more time to solve. These findings contribute to a better understanding of the interaction between System 1 and System 2 at the metacognitive and cognitive levels while performing the problem solving task.

According to our view, this study also allows a more nuanced analysis of the interrelations between variables and particularly the capacity of the CRT to predict verbal relational reasoning (DRT) and visual relational reasoning (RPMT). The proposal that the CRT functions as a measure of analytic System 2 processes supports our hypothesis about its predictive capacity over the DRT and the Raven's test. As noted in the Introduction, these two tests assess verbal and visual abstract reasoning, respectively, and require thinking in a reflective, controlled and supervised way. The study provides new novel findings regarding the positive relation of cognitive reflection with respect to reasoning. It also extend a number of previous findings that showed that there is a moderate correlation between CRT and other intelligence measures, such as Wonderlic IQ test (Frederick, 2005; Toplak et al., 2011).

The results found in DRT show a pattern of increased difficulty, the most difficult of which was the syllogistic deductive task and the easiest the syllogistic metadeductive task. This pattern of results can be explained with reference to two main variables. The first variable is the kind of task participants are required to complete. This involves two sources of difficulty: whether the task is deductive or an easier metadeductive task; and whether the task is a generation or an easier evaluation task. The second variable is the number of models required to solve each problem, as predicted by mental models theory (Johnson-Laird and Byrne, 2002): the present results confirm the well-established findings indicating that inferences that call for multiple models are harder than those that call for a single model.

We also investigated the relation between the three reasoning measures and academic achievement measured by means of teacher grades. The inter-correlations between reasoning measures and academic achievement were again in the predicted direction, reliable and moderate. These correlations are somehow lower than the correlations usually found between intelligence and standardized test of achievement (see, for instance, Deary et al., 2007; Hannon, 2016), but they are quite similar to the correlations found by Soares et al. (2015) by using teachers′ assessments of academic performance and the Reasoning Tests Battery (abstract, verbal, numerical, mechanical and spatial reasoning subtests). Likewise, the findings are informative because they are consistent with those of previous studies (e.g., Frederick, 2005; Obrecht et al., 2009) that showed moderate and positive correlations between CRT and SAT (Scholastic Assessment Test) scores, one of the most used measures of academic achievement. The results also extend those of Gómez-Chacón et al. (2014) showing the interaction between cognitive reflection, WM, reasoning and mathematical achievement (i.e., academic qualifications) in secondary school. However, the highest correlations found in the present study were those between Academic Achievement and Deductive Inferences. This result is particularly relevant, especially if we notice that it is higher than the correlation found with fluid intelligence as measured by the RPMT.

The second main aim concerns the predictive ability of the reasoning measures on academic achievement. In that respect, we found that the reliable variables were intelligence and deductive reasoning for math achievement, whereas only deductive reasoning was for global academic achievement. The findings are in accordance with those of previous studies that using different methodological approaches evidenced that intelligence is an important predictor of achievement and rate of learning (e.g., Primi et al., 2010; Roth et al., 2015), either when intelligence is measured by Wechsler Intelligent Scales for Children (Miranda et al., 2012) or Kaufman Test of Cognitive Ability (Kaufman et al., 2012; Scheiber, 2015). Likewise, our results corroborate findings that showed that individual differences in fluid intelligence are strongly related to math achievement (e.g., Primi et al., 2013), confirming thus the high ability of intelligence to predict achievement, particularly mathematics achievement (Primi et al., 2013) over achievement in more language based subjects (Deary et al., 2007). Regarding the predictive effect of reasoning, our results stress the relevance of deductive reasoning as predictor of academic outcomes, in line with previous results. For instance, Bhat (2016) reported that, out of six dimensions of reasoning (i.e., inductive, deductive, linear, conditional, cause-and-effect and analogical reasoning components) assessed on 10th grade students, the maximum involvement was reflected by deductive reasoning followed by cause-effect reasoning and inductive reasoning.

Nevertheless, the most interesting and new finding is that a measure of verbal deductive reasoning predicts Academic Achievement better, even in mathematics, than a visual fluid intelligence measure. As a matter of fact, the main source of novelty is the use itself of an abstract verbal measure of reasoning to predict academic achievement. Oral and written verbal communication is clearly the main way teachers and learners interact and the main source of knowledge acquisition at school. Furthermore, mathematical reasoning and problem solving has a verbal component (see Kintsch and Greeno, 1985), and different studies have confirmed the predictive capacity of reading abilities on mathematics (see García-Madruga et al., 2014b). Our deductive problems involve not only reading comprehension but also, as Raven problems, abstract relational reasoning. Therefore, these results are not entirely unexpected and should not surprise us. The acquisition of new and complex knowledge by means of declarative learning across the various subjects at school relies on basic oral and written communicative abilities, and visual and verbal abstract reasoning.

There might be many factors that have an influence and can also explain the variation in academic achievement. Thus, even though the prediction of academic performance has been widely explored and well-established in relation to cognitive factors such as intelligence or basic cognitive processes, there is a broad consensus that multiple cognitive, personality and motivational variables contribute in an interrelated form to predict individual differences in academic achievement (see, e.g., Ackerman and Heggestad, 1997; Chamorro-Premuzic and Furnham, 2008). For instance, personality predicts academic performance, even when intelligence and cognitive factors are controlled in adolescents (Noftle and Robins, 2007; Leeson et al., 2008); academic motivation (Lee and Shute, 2010), attitudes (Vilia et al., 2017), approaches to learning, prior knowledge, study time, homework parents ′education level are also associated to academic achievement (Núñez et al., 2014). Therefore, student related factors, school environment and peer influences not studied here should also be included to better assess students learning in future research.

The current study has some limitations that should be addressed in future studies. In that respect, we should mention the small sample size. The generalization of our results to the wider population of 15–17 years old adolescents pose some difficulties, due not only to the scarce number of participants but also to the special characteristics of our sample: all of them were science students. Hence, our initial results are thus needed for further confirmation by means of studies using a wider sample of students. Reasoning ability appears to be important for predicting scholastic achievement in sciences, whereas other subjects such as languages seem to be more affected by gender-related attributes. Because our study does not seek to examine gender effects and due our preliminary analyses of data showed no gender differences in DRT and academic achievement measures, we did not consider the effect of such variable in the entire sample of the current study. However, gender differences in scholastic achievement as mediated by reasoning ability should be considered as an improvement to be undertaken in future research (see Kuhn and Holling, 2009).

### Educational implications and conclusion

Our research has some evident applications to educational practice. First, it stresses that the evaluation of students' cognitive abilities at school should not only be based on standard intelligence tests. In this line, Lee and Shute (2010) and Soares et al. (2015) have emphasized the relevance of a multidimensional assessment of students' cognitive abilities. Without undermining the basic role of intelligence tests at school, our results confirm this approach that highlights that verbal deductive and cognitive reflection abilities might be as important at school as standard intelligence tests. As Toplak et al. (2011) argued, the CRT captures important characteristics of rational thinking that are not measured in other intelligence tests. Both reasoning tasks, CRT and DRT, may be considered by teachers and educational psychologists as a relevant source of information to infer about subsequent school success/failure or to diagnose learning difficulties. Second, our research confirms the importance of a dual-processes approach in education. CRT provides a measure of student propensity to be reflexive and resist intuitive responses when faced with solving quite difficult, even if apparently easy, intellectual problems. As all good teachers are aware, a deeper understanding of concepts and tasks, as well as the inhibition of superficial processes and responses, are crucial in education. CRT allows us to know which students are probably going to need specific attention to grow more reflective and think in a deeper way in their leaning. A third educational corollary of our research is the relationship between metacognitive factors and academic achievement. Two of our measures, CRT and DRT, include an assessment of participants' metacognitive abilities: the ratings of difficulty in CRT and the metacognitive inferences in DRT. The correlations of these measures with both academic measures confirm the important role of metacognitive issues in education and underline again the required introduction of metacognition in ordinary teaching and learning at school. Finally, the study of individual differences in reasoning measured by the three reasoning tasks is useful to understand better how instruction can be made more effective for more learners. Attempts are being made to elucidate the role of reasoning skills so that appropriate interventions to foster students′ reasoning abilities and preventive strategies can be identified (e.g., Ariës et al., 2014). From the perspective of dual processes in reasoning, the fact that System 2 entails conscious reasoning makes it susceptible to educational intervention. The promotion of in-depth understanding and the inhibition of superficial processes and strategies that otherwise lead to error would be considered as relevant instructional strategies.

To end, complex declarative –meaningful- learning at school is a kind of human thinking activity that involves the building of mental representations by integrating external and previously stored information, and their manipulation in WM. Along with other higher-order thinking abilities, meaningful learning is a complex and sequential task that demand learners to activate and use executive functions. In this paper we have presented some preliminary evidences confirming that the presumed outcome of meaningful learning (i.e., academic achievement) in diverse subjects is tightly related to three kinds of abstract reasoning: cognitive reflection, verbal deduction and intelligence. Cognitive reflection provides a measure of executive functioning and therefore underlies reasoners' performance in verbal and visuo-spatial reasoning tasks. Likewise, the two new measures of abstract reasoning and the classic measure of intelligence are able to predict a substantial amount of variance across the main academic performance measures. Finally, our results confirm the importance of dual processes, verbal deductive and metacognitive approaches in ordinary teaching and learning at school. By this means, the study will provide valuable information about the possible evaluation and intervention by educational psychologist.

## Ethics statement

This study was carried out in accordance with the recommendations of UNED ethics committee with written informed consent from legal tutors of all subjects. All legal tutors gave written informed consent in accordance with the Declaration of Helsinki. The study and overall procedure were approved by the UNED ethics committee.

## Author contributions

IG-V and JG, Principal Investigators of the R&D Project, substantially contributed to the writing of the manuscript, which was revised critically by JV and GD. All authors contributed to all steps and approved the final version of the manuscript. The authors agree to be accountable for all aspects of the study in ensuring that questions related to the accuracy or integrity of any part of the work are appropriately investigated and resolved.

### Conflict of interest statement

The authors declare that the research was conducted in the absence of any commercial or financial relationships that could be construed as a potential conflict of interest.
